# Features of Activity of the Phenylpropanoid Biosynthesis Pathway in Melanin-Accumulating Barley Grains

**DOI:** 10.3389/fpls.2022.923717

**Published:** 2022-07-11

**Authors:** Anastasiia Y. Glagoleva, Alexander V. Vikhorev, Nikolay A. Shmakov, Sergey V. Morozov, Elena I. Chernyak, Gennady V. Vasiliev, Natalia V. Shatskaya, Elena K. Khlestkina, Olesya Y. Shoeva

**Affiliations:** ^1^Institute of Cytology and Genetics, Siberian Branch of the Russian Academy of Sciences, Novosibirsk, Russia; ^2^Kurchatov Genomics Center, ICG, SB RAS, Novosibirsk, Russia; ^3^N.N. Vorozhtsov Novosibirsk Institute of Organic Chemistry, SB RAS, Novosibirsk, Russia; ^4^N.I. Vavilov All-Russian Research Institute of Plant Genetic Resources, Saint Petersburg, Russia

**Keywords:** benzoic acid, hydroxycinnamic acid, *Hordeum vulgare*, metabolome, phenylpropanoids, transcriptome

## Abstract

Barley (*Hordeum vulgare* L.) grain pigmentation is caused by two types of phenolic compounds: anthocyanins (which are flavonoids) give a blue or purple color, and melanins (which are products of enzymatic oxidation and polymerization of phenolic compounds) give a black or brown color. Genes *Ant1* and *Ant2* determine the synthesis of purple anthocyanins in the grain pericarp, whereas melanins are formed under the control of the *Blp1* gene in hulls and pericarp tissues. Unlike anthocyanin synthesis, melanin synthesis is poorly understood. The objective of the current work was to reveal features of the phenylpropanoid biosynthesis pathway functioning in melanin-accumulating barley grains. For this purpose, comparative transcriptomic and metabolomic analyses of three barley near-isogenic lines accumulating anthocyanins, melanins, or both in the grain, were performed. A comparative analysis of mRNA libraries constructed for three stages of spike development (booting, late milk, and early dough) showed transcriptional activation of genes encoding enzymes of the general phenylpropanoid pathway in all the lines regardless of pigmentation; however, as the spike matured, unique transcriptomic patterns associated with melanin and anthocyanin synthesis stood out. Secondary activation of transcription of the genes encoding enzymes of the general phenylpropanoid pathway together with genes of monolignol synthesis was revealed in the line accumulating only melanin. This pattern differs from the one observed in the anthocyanin-accumulating lines, where — together with the genes of general phenylpropanoid and monolignol synthesis pathways — flavonoid biosynthesis genes were found to be upregulated, with earlier activation of these genes in the line accumulating both types of pigments. These transcriptomic shifts may underlie the observed differences in concentrations of phenylpropanoid metabolites analyzed in the grain at a late developmental stage by high-performance liquid chromatography. Both melanin-accumulating lines showed an increased total level of benzoic acids. By contrast, anthocyanin-accumulating lines showed higher concentrations of flavonoids and *p*-coumaric and ferulic acids. A possible negative effect of melanogenesis on the total flavonoid content and a positive influence on the anthocyanin content were noted in the line accumulating both types of pigments. As a conclusion, redirection of metabolic fluxes in the phenylpropanoid biosynthesis pathway occurs when melanin is synthesized.

## Introduction

**FIGURE 1:**
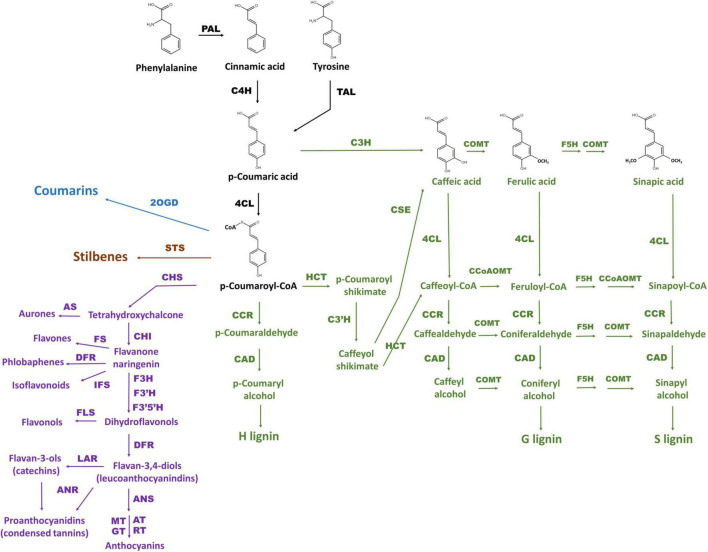
The scheme of phenylpropanoid biosynthesis including pathway branches for flavonoids (purple), lignins (green), stilbenes (brown), and coumarins (blue) according to Deng and Lu (2017) and Marchiosi et al. (2020). General phenylpropanoid pathway reactions are black. The enzymes are PAL: phenylalanine ammonia lyase, C4H: cinnamate 4-hydroxylase, 4CL: 4-coumarate CoA ligase, HCT: hydroxycinnamoyl CoA:shikimate hydroxycinnamoyl transferase, C3H: *p*-coumaroyl shikimate 3-hydroxylase, C3′H: 4-coumaroylshikimate/quinate 3′-hydroxylase, CSE: caffeoyl shikimate esterase, CCoAOMT: caffeoyl CoA O-methyltransferase, F5H: ferulate 5-hydroxylase, COMT: caffeic acid O-methyltransferase, CCR: cinnamoyl CoA reductase, CAD: cinnamyl alcohol dehydrogenase, CHS: chalcone synthase, CHI: chalcone-flavanone isomerase, AS: aureusidin synthase, F3H: flavanone 3-hydroxylase, FLS: flavonol synthase, FS: flavone synthase, F3′H: flavonoid 3 / c-hydroxylase, F3′5′H: flavonoid 3 / c,5 / c-hydroxylase, IFS: isoflavonoid synthase, DFR: dihydroflavonol 4-reductase, ANS: anthocyanidin synthase, AT: acetyltransferase, GT: glycosyltransferase, MT: methyltransferase, RT: rhamnosyltransferase, LAR: leucoanthocyanidin reductase, ANR: anthocyanidin reductase, STS: stilbene synthase, 2OGD: 2-oxoglutarate-dependent dioxygenase.

Phenylpropanoids are naturally occurring phenolic compounds. They represent a very diverse class of plant secondary metabolites, derived from phenylalanine, and play a vital role in plant physiology. These compounds are essential structural components of cell walls, protect plants against various biotic and abiotic environmental factors, act as phytoalexins against herbivores and pathogens, and—because some of them are pigments—mediate interactions of plants with pollinators and seed dispersers (Treutter, 2006; Vogt, 2010; Kiani et al., 2021; Gharaghanipor et al., 2022). Synthesis of all phenylpropanoids starts with the conversion of phenylalanine (or tyrosine) originating from the shikimate pathway by phenylalanine ammonia lyase (PAL; or tyrosine ammonia lyase, TAL), cinnamate 4-hydroxylase (C4H), and 4-coumaroyl coenzyme A (CoA) ligase (4CL) into *p*-coumaroyl-CoA, which is a precursor for all subsequent pathway branches and resulting metabolites including benzoic acids, flavonoids, monolignols, stilbenes, and coumarins (Vogt, 2010; Deng and Lu, 2017; Figure 1). Discovery of *p*-coumaric acid as a monomer of the melanin accumulating in oat bracts, allows to categorize melanin as a phenylpropanoid too (Varga et al., 2016).

Melanin is a widespread pigment in plant kingdom. It accumulates mostly in covering tissues of seeds, where it defends embryo against excess solar radiation, mechanical injury, and dehydration (Riley, 1997; Pandey and Dhakal, 2001; Fritz and Saukel, 2011; Lusa et al., 2018). Due to the unique features of melanin that were revealed by studies of animal, fungal and synthetic melanins, perspectives to use it in a broad range of biomedical and technological applications has been demonstrated (d’Ischia et al., 2015; Di Mauro et al., 2017; Vahidzadeh et al., 2018). Recently sunflower husks melanin was successfully used as a sorbent with high enterosorption efficiency and as an antiaging agent in elastomer compositions (Gracheva and Zheltobryukhov, 2019; Kablov et al., 2019). Besides technological application, melanin is considered as a food supplement that can increase nutrition value of stable food, for example, the melanin isolated from black garlic (Wang and Rhim, 2019). The implementation of melanin powder obtained from buckwheat husks into the desserts increased their antioxidant activity (Korpacheva et al., 2021). Despite the important functions of this pigment for plants and perspectives of its broad applications, it remains one of the poorly studied pigment in plants.

Melanin from different sources is a high-molecular-weight black-and-brown pigment that is formed by a common mechanism, which includes two stages: the oxidation of phenolic compounds by polyphenol oxidases (PPOs) and subsequent polymerization of the resultant intermediates (Britton, 1983). In plants, this process is also known as the enzymatic browning reaction, which takes place in damaged plant tissues (Nicolas et al., 1994). Participation of PPOs in melanogenesis in intact plant tissues was proposed recently (Boeckx et al., 2017), and genes encoding these enzymes have been shown to be involved in melanin synthesis in the seeds of rice, peanut, sesame, and watermelon (Fukuda et al., 2012; Wan et al., 2016; Li et al., 2020; Wang et al., 2020).

Plant melanin represents a heterogeneous group of compounds because different precursors can participate in its synthesis, among which catechol and caffeic, chlorogenic, gallic, *p*-coumaric, and protocatechuic acids have been reported (Solano, 2014; Varga et al., 2016; Jukanti, 2017). Identification of substrates for PPOs in a given plant species is considered the main difficulty with assigning melanin synthesis to biosynthetic pathways. Nevertheless, given that the same substrates can be used for the synthesis of melanin and of other phenylpropanoids, redirection of a biosynthetic pathway and as a consequence some metabolic shifts in the metabolism of phenolic compounds can be hypothesized when melanin is being produced in an organism. Research into such metabolic shifts makes it possible to trace the pathways involved in melanin formation. As a genetic model for such study the barley (*Hordeum vulgare* L.) was chosen.

The grain of this plant species can be colored by pigments of a phenolic nature: anthocyanins (which are flavonoids) give a purple and blue color, and melanin bestows a black or brown color to the grain. Synthesis of these pigments is inherited independently: genes *Ant1* and *Ant2* determine the biosynthesis of purple anthocyanins in the pericarp (Gordeeva et al., 2019), *Blx1–5* control the biosynthesis of blue anthocyanins in the aleurone layer (Strygina et al., 2017), whereas melanin is produced under the control of the *Blp1* gene in hulls and the pericarp (Costa et al., 2001; Long et al., 2019). Simple genetic control of anthocyanin and melanin synthesis in barley and the availability of backcrossed near-isogenic lines (NILs) differing in dominant alleles of *Ant1*, *Ant2*, and *Blp1* (Druka et al., 2011), make barley a convenient genetic model for exploring metabolic and transcriptomic differences underlying melanogenesis.

Although precursors of barley melanin have not been identified yet, transcriptional activation of the gene encoding PAL, but not the genes encoding enzymes of the flavonoid branch of the pathway, in a melanin-accumulating NIL (Glagoleva et al., 2017) suggests that phenolic substrates synthesized prior to the branching of the phenylpropanoid pathway may participate in melanin synthesis. In particular, an effect of the *Blp1* locus on phenylpropanoid metabolism was revealed in a barley NIL accumulating anthocyanin and melanin pigments in the grain (and carrying dominant alleles of *Ant1*, *Ant2*, and *Blp1*). In this line, earlier activation of anthocyanin synthesis and a higher content of anthocyanins in the grain were registered in comparison to the line that accumulates anthocyanins only (*Ant1*, *Ant2*) (Glagoleva et al., 2022).

The objective of the current work was to reveal further features of the phenylpropanoid biosynthesis pathway functioning in melanin-accumulating barley grains and the effects of the *Blp1* locus on phenylpropanoid metabolism. For this purpose, comparative transcriptomic and metabolomic analyses of three barley NILs accumulating anthocyanins, melanins, or both in the grain were performed.

## Materials and Methods

### Plant Material and Phenotyping

Three NILs obtained in the spring *cv*. Bowman (“Bowman From Fargo,” NGB22812)^1^ genetic background was used in the study. The first one is the i:Bw*Blp1* line (NGB20470; hereafter: BLP – black lemma and pericarp) carrying the *Blp1* locus mapped to chromosome 1HL (Druka et al., 2011) and characterized by melanin accumulation in hulls and the grain pericarp. The second NIL is i:Bw*Ant1Ant2* (NGB22213; hereafter: PLP – purple lemma and pericarp), which is characterized by anthocyanin accumulation in the grain pericarp and leaf sheath and carries complementary genes *Ant1* and *Ant2* located on chromosomes 7HS and 2HL, respectively (Druka et al., 2011). The third NIL is the i:Bw*Ant1Ant2Blp1* line (hereafter: BP – black and purple), which was developed previously via marker-assisted selection on the basis of lines BLP and PLP and is characterized by the accumulation of both anthocyanins and melanins in the grain (Glagoleva et al., 2022). The plants were grown in a greenhouse of the Institute of Cytology and Genetics (ICG) SB RAS (Novosibirsk, Russia) under a 12 h photoperiod in a temperature range of 20–25°C.

### Extraction and Chromatographic Analysis of Phenylpropanoids

To analyze the phenylpropanoid content of the grains, three NILs (BLP, PLP, and BP) and *cv.* Bowman (parental line) were collected at the early dough stage (BBCH-83) of spike maturity. The whole grains were frozen in liquid nitrogen and dried in vacuum. Then, the dried grains were ground up and used for phenolic-compound extraction. Phenolic compounds (phenolic acids, flavonoids, and anthocyanins) were extracted from 300 mg of mature seeds with 2.5 mL of a methanol:2% formic acid mixture (7:3, v/v) (Shoeva et al., 2016) with ultrasonication at 40°C. Three independent extraction procedures were performed for each genotype under study. After centrifugation, the extracts were analyzed with Agilent 1100 liquid chromatography system (Agilent Technologies, Santa Clara, CA, United States) equipped with a quaternary pump, online degasser, autosampler, and diode array detector. Separation was implemented on an Eclipse SB-C18 (4.6 mm × 150 mm, 5 μm) column in a binary solvent system consisting of 0.1% CF_3_COOH in water and methanol. The gradient of methanol was linear from 2 to 98% for 40 min, the injection volume was 4 μL, the column temperature was 23 ± 2°C, the flow rate was 0.8 mL/min. The high-performance liquid chromatography (HPLC) runs were monitored at 254 nm for benzoic acids, at 320 nm for cinnamic acids, at 360 nm for flavonoids, and at 520 nm for anthocyanins. Identification and quantification of benzoic and cinnamic acids, flavonoids, and anthocyanins were based on comparisons of the UV data and HPLC retention times of standard compounds. The limit of detection (LOD) was 0.2 μg/g in sample and the limit of quantification (LOQ) was 0.5 μg/g in sample.

### Chemicals and Reagents

The following reagents were used as a standard compounds: 3,4-dihydroxybenzoic acid, catechin, dihydromyricetin, vanillic acid, *trans*-caffeic acid, 4-hydroxycinnamic acid, dihydroquercetin, *trans*-ferulic acid, dimethoxybenzoic acid, 3-hydroxycinnamic acid, dihydrokaempferol, hesperidin, rutin, trihydrate, 2-hydroxycinnamic acid, cinnamic acid, luteolin, apigenin, quercetin, naringenin, 3-*O*-glucoside cyaniding, 3-glucoside malvidin (Merck, Darmstadt, Germany).

### RNA Extraction and Sequencing

**FIGURE 2:**
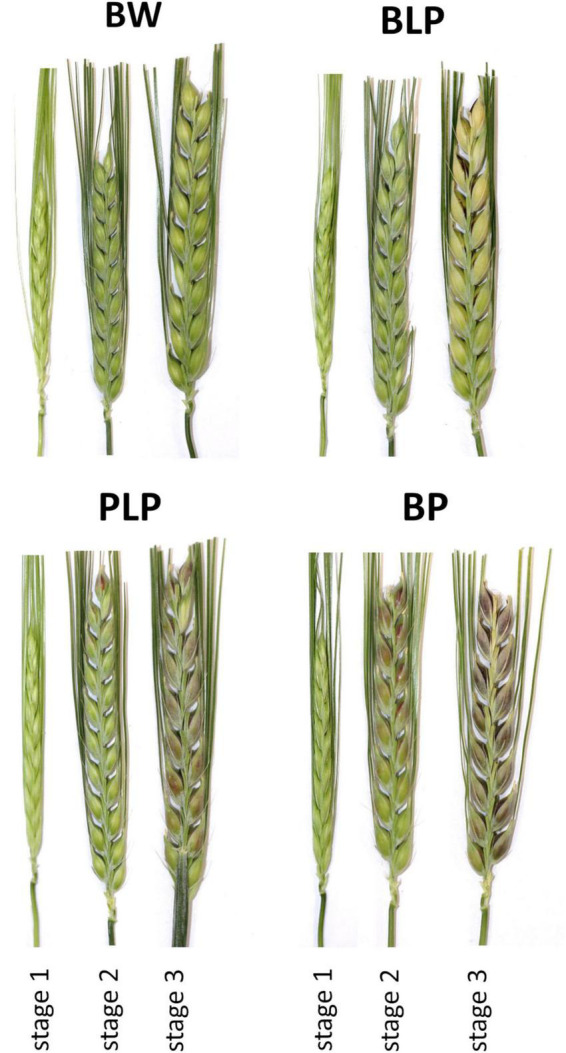
Spikes of barley NILs BLP, PLP, BP, and of cv. Bowman (BW) used in the study at the booting stage (1), late milk stage (2), and early dough stage (3).

For a comparative high-throughput RNA sequencing (RNA-seq) analysis, total RNA was isolated from the lines BLP, PLP, BP, and parent *cv.* Bowman at three stages of spike development. The stage 1 is the booting stage, when first awns are visible (BBCH-49); stage 2 is the late milk stage (BBCH-77), when the start of anthocyanin pigmentation is observed; and the stage 3 is the early dough stage (BBCH-83), when melanin pigmentation appears. At the stage 1, RNA was extracted from fresh whole flowers, which was separated from the spike and homogenized in liquid nitrogen. At the stages 2 and 3, pericarp and hulls were peeled from fresh grains of the NILs and homogenized in liquid nitrogen. Spikes of near-isogenic lines under study are presented in Figure 2. RNA was extracted in three biological replicates using the RNeasy Plant Mini Kit (Qiagen, Hilden, Germany); each replicate combined material from three plants (i.e., nine plants of each line were analyzed in total as three biological replicates). After the removal of DNA traces with the Turbo DNA free kit, RNA quality was assessed using Bioanalyzer 2100 with the RNA Nano kit. An mRNA fraction was isolated, and barcoded RNA-seq libraries for the Illumina system were generated by means of TruSeq Stranded mRNA Library Preparation Kit A. The resulting 36 barcoded libraries (3 biological replicates × 4 barley lines × 3 stages of development) were sequenced on an Illumina NextSeq 550 instrument with the NextSeq 500 High Output v2 Kit (75 cycles).

### RNA-Seq Data Analysis

FASTQC v.0.11.9 was employed to evaluate the libraries’ quality (Andrews, 2010). Filtering of the libraries was performed in Trimmomatic software v.0.39 with the following parameters: “LEADING:20 TRAILING:20 SLIDINGWINDOW:4:20 MINLEN:50” (Bolger et al., 2014). This approach results in the removal of unidentified bases (N) or bases with a Phred quality score below 20 from both 3′ and 5′ ends of a read, in the removal of reads with length less than 50, and in the trimming of 5′ ends of the read when mean quality in the sliding window of length 4 drops below 30. DART tool v.1.4.2 (Lin and Hsu, 2018) was used to align the filtered reads to barley genome assembly IBSC v.2 (Mascher et al., 2017) release 47 from the Ensembl Plants database^2^. The reads aligned to each gene were counted with the help of the featureCounts function in the Subread software (Liao et al., 2014). Based on the obtained counts, principal component analysis was conducted using the DESeq2 function of plotPCA (Love et al., 2014). Raw read counts data before normalization and genes expression after CPM (counts per million mapped reads) normalization are presented in Supplementary Data Sheets 1, 2.

Differential gene expression analysis was performed by means of the edgeR package for R (Robinson et al., 2010). Weakly expressed genes were eliminated using the “filterByExpr” function. Differential expression between samples was detected via the generalized linear model approach. Differential expression was calculated between time stages within each barley line and between barley lines within each time stage. Thus, 26 comparisons were performed. Genes with a false discovery rate (FDR) of <0.05 and an absolute value of a log_2_-transformed fold change greater than 2 (| log_2_(FC)| > 2) were considered differentially expressed.

For functional annotation of differentially expressed genes (DEGs), Gene Ontology (GO) enrichment analysis and metabolic pathway involvement analysis were undertaken. Singular enrichment analysis of DEG lists was performed using the AgriGO v.2 toolkit (Tian et al., 2017). For each comparison performed for the differential expression analysis, lists of upregulated and downregulated genes were examined separately. GO terms with adjusted *p*-value < 0.05 were regarded as significantly enriched. KEGG pathways enriched in the DEG lists were identified in BlastKoala v.2.2 (Kanehisa et al., 2016). In this case, amino acid sequences of DEGs were subjected to a BLAST search against a non-redundant dataset of pangenome sequences at the genus level and then annotated in KEGG Ontology (KO) terms by the KOALA algorithm. Barley genes involved in the phenylpropanoid biosynthesis pathway were detected via a homology search in the BioMart software using *Arabidopsis thaliana* genes participating in the phenylpropanoid biosynthesis pathway (KEGG id: KO00940) as a query. Heatmap figures were generated using the seaborn v.0.11.2 library in Python.

### Verification of the RNA-Seq Data by Quantitative Reverse-Transcription PCR

Nine genes with different expression patterns were chosen randomly for the qRT-PCR verification of the RNA-seq data: *HORVU1Hr1G011930, HORVU2Hr1G103040, HORVU2Hr1G103000, HORVU3Hr1G077790, HORVU4Hr 1G090870, HORVU6Hr1G075900, HORVU3Hr1G077960, HORVU7Hr1G098280*, and *HORVU4Hr1G014010*. The qRT-PCR was performed on a QuantStudio 5 machine (Applied Biosystems, Waltham, MA, United States)^3^ with the HS-qPCR Lo-ROX SYBR kit (Biolabmix, Novosibirsk, Russia) in a 15 μL reaction mixture. The number of amplification cycles and annealing temperature were optimized for each primer pair (Supplementary Table 1). Three technical replicates of each reaction were run. Gene expression levels were calculated by the relative-standard-curve method and normalized to the geometrical mean of *actin* and *ubiquitin* expression (Himi et al., 2005; von Zitzewitz et al., 2005).

## Results

### Transcriptome Sequencing and Identification of Differentially Expressed Genes

A total of 36 mRNA libraries were sequenced that were derived from grain envelopes of the three NILs (BLP, PLP, and BP) and parental *cv.* Bowman at three stages of spike development: stage 1, booting; stage 2, late milk; and stage 3, early dough (three biological replicates for each type of sample). In total, 801,184,069 single-end 75 bp raw reads were obtained. After filtering, 741,311,996 (92.51%) reads were retained. On average, 92.43% (71.11% unique) reads were successfully mapped to the reference barley genome in the DART software (Supplementary Table 2).

**FIGURE 3:**
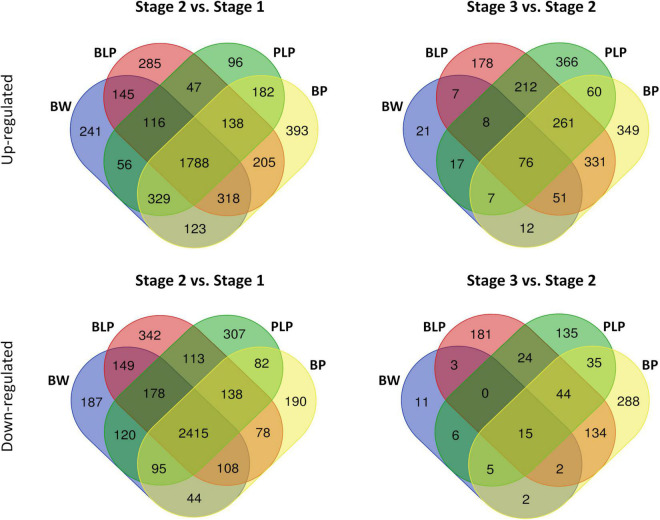
Venn diagrams of upregulated and downregulated DEGs between stages 1 and 2 and between stages 2 and 3 within the lines.

After the removal of genes with low expression and CPM (counts per million mapped reads) normalization, there were 30,969 genes with an expression level higher than the significance threshold. Then, differential expression was calculated between stages within each line and between lines within each stage (Supplementary Table 3). An average, the number of upregulated and downregulated DEGs between the NILs at the stage 1 were 23 and 54, respectively, at the stage 2 – 283 upregulated and 130 downregulated DEGs, and at the stage 3 – 677 upregulated and 304 downregulated DEGs between the NILs under study were revealed (Supplementary Figures 1–3). In a comparison of numbers of DEGs between stages 1 and 2 within the lines, on average 1,788 upregulated and 2,415 downregulated DEGs that are common to all lines were found. The average number of line-specific DEGs was approximately an order of magnitude less than the number of common genes (Figure 3). In contrast, 76 upregulated and 15 downregulated DEGs that are common for all lines were revealed between stages 2 and 3, whereas the number of line-specific DEGs was substantially greater in all lines characterized by pigment accumulation but not in the uncolored parental line (Bowman). It is possible that at the early stages of spike development, genes responsible for plant primary metabolism that are common to all the tested lines were activated regardless of grain pigmentation; on the contrary, during spike maturation, genes of special or secondary metabolism may be activated, which is associated with pigment synthesis in the barley grain.

### Gene Ontology Term Enrichment in Lists of Differentially Expressed Genes

The lists of DEGs between different lines and different stages were annotated by singular enrichment analysis of GO terms to gain a better understanding of the DEGs functions. In total, 49.4% of upregulated genes and 52.2% of downregulated genes were annotated successfully. Full lists of DEGs along with GO term enrichment are presented in Supplementary Table 4.

**FIGURE 4:**
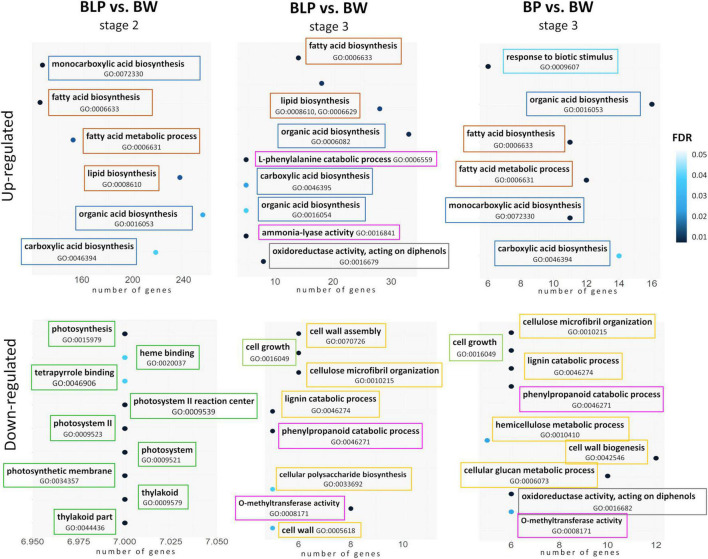
GO term enrichment within lists of upregulated and downregulated DEGs in lines BLP and BP in comparison with Bowman (BW). Related metabolic processes, functions, and compartments are highlighted in the same color.

At the booting stage (stage 1), no significantly enriched GO terms were found between the non-pigmented Bowman cultivar and the lines characterized by accumulation of melanins and/or anthocyanins. In contrast, there were differences between the lines in metabolic processes in conjunction with pigmentation at the later stages. In the lines accumulating melanins (BLP and BP), the lists of upregulated DEGs at stages 2 and 3 were found to be enriched with GO terms related to fatty acid biosynthesis, lipid biosynthetic process, and organic acid biosynthesis when compared with Bowman. On the other hand, the upregulation of genes involved in the L-phenylalanine metabolic process and ammonia-lyase activity was detectable only in the BLP line at the later stage of spike maturation (stage 3) in comparison with Bowman (Figure 4). In the list of downregulated DEGs, GO terms “photosynthesis,” “thylakoid,” and “photosystem” were found for the BLP line at stage 2, but they were absent in the other pigmented lines in comparison with Bowman. Moreover, the genes involved in cell wall biogenesis and assembly, lignin metabolic process, and cell growth turned out to be downregulated in lines BLP and BP when compared with Bowman at stage 3 but not in the PLP line. In the comparison of the PLP line with Bowman, there were no line-specific GO terms that were not detectable in the other lines.

### Identification of Genes Involved in Phenylpropanoid Biosynthesis

**FIGURE 5:**
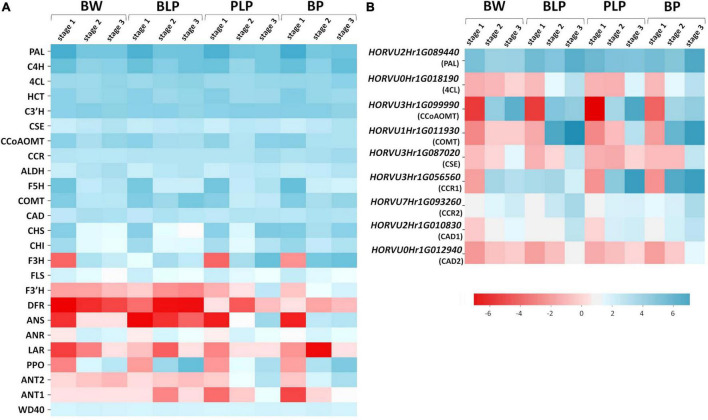
Heatmap analysis of mean expression levels of the key genes of phenylpropanoid biosynthesis **(A)** and the genes with line-specific expression **(B)** in lines Bowman (BW), BLP, PLP, and BP at the three stages of spike development.

Barley genes encoding the main enzymes of the phenylpropanoid biosynthesis pathway were identified in the KEGG database via homology with *A. thaliana* genes (Supplementary Table 5). Most of these enzymes are encoded by gene families in the barley genome; therefore, we used mean levels of gene expression for heatmap construction (Figure 5A). According the expression patterns, the genes can be subdivided into two groups: early and late genes. The early genes are activated at the booting stage (stage 1) in all four lines regardless of pigmentation. These include the following genes: *PAL*, *C4H*, *4CL*, *C3′H*, *CSE*, *CCoAMT*, *COMT*, *F5H*, *CCR*, *CHS*, *CHI*, and *FLS*. The late genes are transcribed at later stages of spike development (stages 2 and 3), and their expression proved to be line-specific and depended on the pigment type accumulated. In lines characterized by the presence of anthocyanins (PLP and BP), specific activation of the flavonoid biosynthesis pathway at the later stages was observed (Figure 5A). The pathway includes enzymes F3H, F3′H, and ANS. Furthermore, secondary activation of genes encoding CHS and CHI, which are implicated in the key steps of flavonoid biosynthesis, was registered in lines PLP and BP. It should be noted that in the BP line, these genes were found to be activated earlier, at stage 2, whereas in BP’s parental line PLP, they are highly transcribed only at stage 3. Moreover, activation of some specific genes from the monolignol branch of the biosynthesis pathway was observed in lines PLP and BP, including gene *HORVU3Hr1G056560* encoding CCR, whereas the expression of gene *HORVU3Hr1G099990* encoding CCoAMT was noticed specifically in the PLP line (Figure 5B).

The expression of regulatory genes *Ant1*, *Ant2*, and *WD40* was investigated too; they are necessary for MBW complex assembly and anthocyanin biosynthesis in the grain pericarp. Specific activation of *Ant1* and *Ant2* was detectable in anthocyanin-accumulating lines; moreover, the expression pattern of the *Ant2* gene was the same as that of the late genes of flavonoid biosynthesis pathway, and activation timing was different between lines PLP and BP. By contrast, the expression of *WD40* was constant in all the lines at all examined stages (Figure 5A).

Similarly, the genes with specific activation in the presence of melanin pigments at stages 2 and 3 were identified in the phenylpropanoid pathway (Figure 5B). They are represented by specific genes from gene families encoding enzymes of the general phenylpropanoid pathway such as PAL (gene *HORVU2Hr1G089440*), and enzymes participating in monolignol synthesis such as COMT (*HORVU1Hr1G011930*), CSE (*HORVU3Hr1G087020*), CCR (*HORVU3Hr1G056560* and *HORVU7Hr1G093260*), and CAD (*HORVU2Hr1G010830* and *HORVU0Hr1G012940*). In addition, genes encoding C4H and 4CL proved to be activated at stage 3 in all pigmented lines; what is more, in the case of *C4H*, there is secondary activation of this gene in response to *Ant1*, *Ant2*, and *Blp1* presence.

Given that melanin is a product of phenolic-compound polymerization, the expression of PPOs was also analyzed. It was observed that PPO genes are inactive at stage 1, and their expression increases during spike maturation in all the analyzed lines, whereas in BLP, these genes are most transcriptionally active (Figure 5A).

### Quantitative Reverse-Transcription PCR Verification of Gene Expression

**FIGURE 6:**
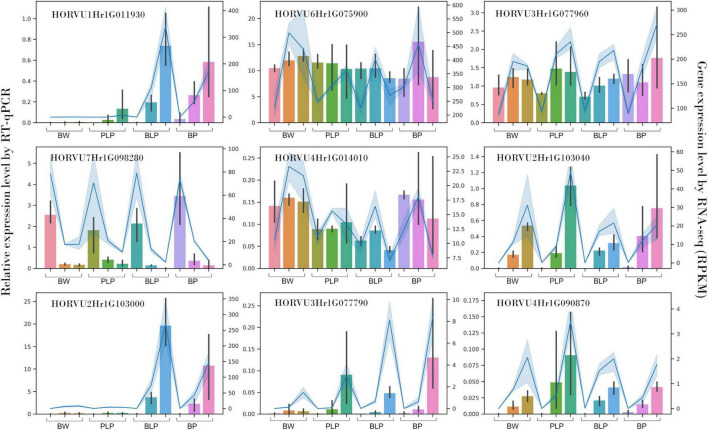
qRT-PCR verification of nine DEGs in NILs (columns) at stages 1, 2, and 3 (left to right) and a comparison with our RNA-seq data (blue line).

Expression of nine genes was confirmed by qRT-PCR. These genes code for o-methyltransferase (*HORVU1Hr1G011930*), polyphenol oxidases (*HORVU2Hr1G103040*, *HORVU2Hr1G103000*, *HORVU3Hr1G077790*, and *HORVU4Hr1G090870*), chloroplastic geranylgeranyl diphosphate reductase (*HORVU6Hr1G075900*), translation initiation factor SUI1 (*HORVU3Hr1G077960*), xyloglucan endotransglucosylase/hydrolase (*HORVU7Hr1G098280*), and chloroplastic photosynthetic NDH subunit of subcomplex B1 (*HORVU4Hr1G014010*). *In vitro* expression analysis of these genes was highly consistent with the *in silico* data (Spearman’s correlation was 0.93), thus indicating the reliability of our RNA-seq data (Figure 6).

### Phenylpropanoid Compounds’ Levels in the Near-Isogenic Lines

**FIGURE 7:**
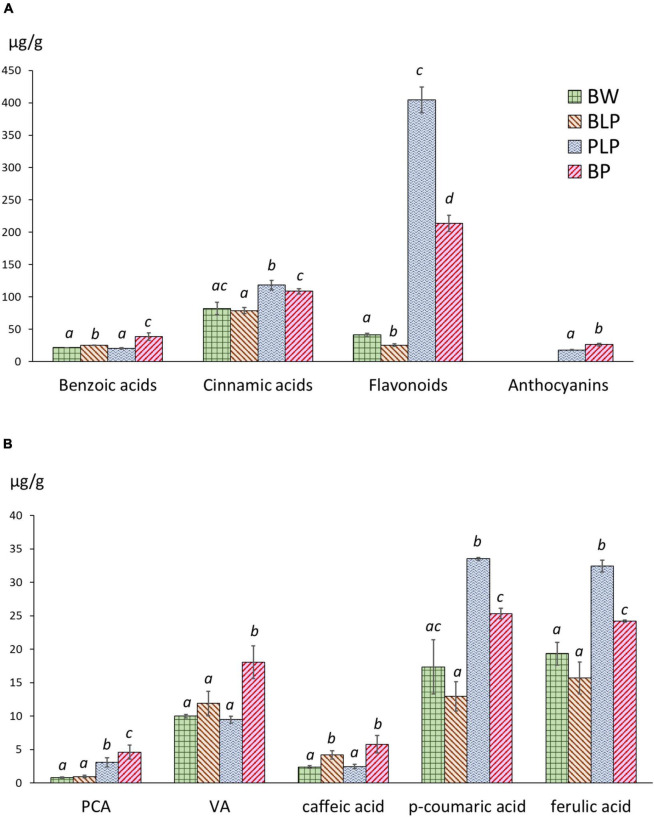
**(A)** The total contents of benzoic acids, hydroxycinnamic acids, flavonoids, and anthocyanins in NILs at the early dough stage (stage 3) of spike maturity. **(B)** Concentrations of individual phenylpropanoids at stage 3. PCA, protocatechuic acid; VA, vanillic acid. The different letters denote statistically significant differences between the lines (*t* test, *p* ≤ 0.05).

Extraction of phenolic compounds with subsequent HPLC analysis was carried out at the 3rd stage of spike development, when the greatest of line-specific differences in gene expression were observed. The quantities of identified phenolic compounds and chromatographic profiles are presented in Supplementary Table 6 and Supplementary Figures 4–7, respectively. The total contents of benzoic acids, hydroxycinnamic acids, flavonoids and anthocyanins are presented in Figure 7A. The total benzoic-acid content was significantly higher in melanin-containing NILs BLP (25.2 ± 0.3 μg/g) and BP (38.6 ± 4.6 μg/g) as compared with PLP (20.6 ± 0.7 μg/g) and *cv.* Bowman (21.5 ± 0.2 μg/g), whereas in the BP line, it was statistically significantly the highest among all the lines. Among the compounds in this family, protocatechuic, 4-hydroxybenzoic, and vanillic acids were identified. It is noteworthy that an elevated concentration of protocatechuic acid was found to be characteristic of lines PLP and BP, which accumulate anthocyanins; a significantly increased content of vanillic acid was also documented in the BP line, whereas in BLP, the increase was not significant (Figure 7B).

The highest level of hydroxycinnamic acids was registered in PLP (118.2 ± 5.9 μg/g); however, this level was also significantly higher in BP (108.7 ± 3.4 μg/g) than in BLP (78.8 ± 3.9 μg/g) and Bowman (82.2 ± 7.8 μg/g). In this family of compounds, caffeic acid, *p*-coumaric acid, *m*-coumaric acid, and ferulic acid were identified. Furthermore, four additional compounds were assigned to the family of cinnamic acids according to their characteristic UV spectra. Two patterns of these acids in the lines under study can be distinguished. The first one concerns caffeic acid accumulation and represents its significantly increased content in NILs with melanin. The second pattern concerns *p*-coumaric and ferulic acids: greater accumulation in lines PLP and BP (Figure 7B).

The flavonoid content in Bowman and BLP was 41.2 ± 2.3 and 25.3 ± 1.7 μg/g, respectively: dramatically different from the flavonoid content in PLP and BP (404.5 ± 16.5 and 213.8 ± 10.3 μg/g, respectively). In this family of compounds, luteolin, apigenin, and quercetin 3-rhamnoglucoside (rutin) were identified. Besides, a series of glycosides of luteolin and apigenin was identified by means of characteristic UV spectra. In the subfamily of luteolin derivatives, a flavonoid was found, which, according to the UV spectrum and retention time, can be luteolin methyl ester. This compound was not found in Bowman and BLP and is one of the major phenolic compounds in lines PLP and BP. Anthocyanins were not detectable in lines Bowman and BLP, but their presence in lines PLP and BP was confirmed. In the family of anthocyanins, cyanidin-3-glucoside and malvidin-3-glucoside were identified. It was noticed that the total concentration of flavonoids was ∼1.5 times higher in PLP than in BP; however, the total anthocyanin content was significantly greater in BP than in its parental line PLP (26.2 ± 1.7 and 18.0 ± 0.4 μg/g, respectively).

## Discussion

Phenolic compounds, including anthocyanin and melanin pigments, that can by synthesized in hulls and pericarp of barley grain, play diverse biochemical and molecular functions in plant physiology (Treutter, 2006; Vogt, 2010; Kiani et al., 2021; Gharaghanipor et al., 2022). They are not only protect the seeds from damage, deterioration, and pest but also are beneficial to human health (Rasouli et al., 2017). Unlike anthocyanins, melanins are a less studied family of pigments. One of obvious reasons for that is the difficulty with their extraction and chemical analysis. To date, to our knowledge, *p*-coumaric acid as a precursor of melanin synthesis has been quantified only in oat bracts (Varga et al., 2016). Here, comparative metabolic and transcriptomic assays of three barley NILs that differ in profiles of anthocyanin and melanin compounds in the grain were carried out to detect metabolic shifts in phenolic compounds including phenylpropanoids and thus identify the pathways that could be involved in melanin synthesis in barley.

Earlier, an increasing total phenolic content was documented in black-grained sesame in comparison with white-grained one (Zhou et al., 2016). By a comparative LC-MS/MS-based metabolome profiles analysis of NaOH and methanol extracts of black and white sesame seeds, benzoic, caffeic, ferulic, homogentisic, indole-carboxylic, protocatechuic, and vanillic acids were identified as the potential precursors of the sesame melanin (Dossou et al., 2022). In the current work taking advantage of precise genetic models, one may state with more confidence that melanogenesis is associated with metabolic shifts of phenolic compounds and with significant alterations in phenylpropanoid metabolism. In barley line BLP, the total benzoic acid content of grain at the soft dough stage of ripeness proved to be higher in comparison with unpigmented-grain Bowman, and addition of the genome fragments of chromosomes 7HS and 2HL carrying, respectively, anthocyanin-specific genes *Ant1* and *Ant2* seemed to increase the benzoic-acid content in the BP line. Among benzoic acids, protocatechuic and vanillic acids were identified. Although the concentration of these compounds was not elevated in the BLP line in comparison with Bowman, the genome fragment of chromosome 1HL carrying *Blp1* probably significantly influences the levels of these compounds in the BP line in comparison with PLP.

Benzoic acids — whose content is elevated in BLP and BP — can be synthesized from intermediates of shikimate or phenylpropanoid pathways (Marchiosi et al., 2020). However, since both melanogenesis and the enzyme which catalyze the enzymatic browning reaction in damaged tissues and are believed to participate in melanin synthesis in intact tissues — PPO — are present in plastids (Boeckx et al., 2015, 2017; Shoeva et al., 2020), the benzoic acids originating from the shikimate pathway that occurs in plastids too are more probable precursors of melanin than compounds originating from the phenylpropanoid pathway that takes place in the cytoplasm (Widhalm and Dudareva, 2015). In barley, substrates for PPOs that could be precursors for melanin synthesis have been identified neither in the grain nor in damaged leaf tissue (Claussen and Pepper, 1968). However, in species related to barley — bread and durum wheat (belonging just as barley to the *Poaceae* family) —, 4-methylcatechol and catechol, respectively, were identified as substrates for PPOs (Jukanti, 2017). Since the nucleotide sequences of the wheats’ PPOs are almost identical to the barley ones (Taketa et al., 2010), one may assume that barley PPOs can use catechol or its derivatives as substrates for melanin synthesis too. Whether barley melanin is consisted of these compounds should be clarified in the future.

Besides changes observed in the content of the simple phenolic compounds (that contain one phenol unit or a derivative of it) in grain at the soft dough stage, the metabolic shifts in the general phenylpropanoid metabolism at transcriptomic level were revealed. At the booting stage, genes of general phenylpropanoid metabolism are activated that are common for all the tested lines regardless of their pigmentation; however, as the spike matures, genes responsible for special metabolism are induced, and the number of DEGs specific for each pigmented line increases. Secondary activation of transcription of genes of the general phenylpropanoid pathway (*Pal*, *C4h*, and *4Cl*) together with monolignol synthesis genes (*Comt*, *Cse*, *Ccr*, and *Cad*), but not flavonoid biosynthesis genes, was observed in the BLP line. This transcription pattern can explain the increased content of caffeic acid in this line, while the total content of flavonoids and anthocyanins turned out to be comparable to these values in Bowman. In contrast, activation of general phenylpropanoid genes *C4h* and *4Cl* together with anthocyanin biosynthesis genes *Chs*, *Chi*, *F3h*, *F3′h*, and *Ans* (occurring earlier in the BP line than in PLP), coincided with upregulation of hydroxycinnamic acids (ferulic and *p*-coumaric), total flavonoids, and anthocyanins in the *Ant1*/*Ant2*-carrying lines in comparison with uncolored Bowman. Given that the total flavonoid content was found to be lower in the BP line than in its parental line PLP, while the anthocyanin content in this line is greater in comparison with PLP, there is possible redirection of the flavonoid biosynthesis pathway when melanogenesis occurs.

Because the lines accumulating either anthocyanin or melanin manifested upregulation of the genes taking part in monolignol biosynthesis at late stages of spike development accompanied by increased concentrations of ferulic and caffeic acids, respectively, one may expect differences in lignin levels between these lines and Bowman. Analysis of the lignin content of barley grains of different colors was performed earlier (Choo et al., 2005). Using diverse genetic materials such as 96 doubled-haploid lines, 40 landraces, four F_3_ bulked populations, and 10 NILs (including those investigated here: Bowman, PLP, and BLP), those authors showed that purple-grained lines do not differ in the lignin content from yellow-grained ones, whereas the black barley is characterized by more protein and a higher lignin content than yellow barley; the three barley samples with the highest concentration of lignin were black seeded (Choo et al., 2005).

Based on these results, we can hypothesize that greater production of benzoic acid in the melanin-accumulating lines may be caused by activation of the shikimate pathway during melanogenesis, which takes place in plastids too. Next, intermediates are introduced into that phenylpropanoid pathway, which is probably redirected either into lignin synthesis in melanin-accumulating lines or into anthocyanin synthesis if anthocyanin-specific regulatory genes are in a dominant state. Whether *Blp1* activates the shikimate pathway (which provides precursors for melanin synthesis and phenylpropanoid pathways) should be clarified in the future.

Besides effect of melanogenesis on phenylpropanoid metabolism, downregulation of genes associated with photosynthesis in stage 2 was revealed in the BLP line compared to Bowman. This finding is consistent with a recent report on oat bracts where the inhibition of photosynthesis was demonstrated at the flowering stage during melanin pigmentation development (Liu et al., 2021). In barley, underexpression of the genes related to photosynthesis was observed in the study at the late milk stage, before appearance of melanins in the grain. Because melanins are synthesized in aging chloroplasts (Shoeva et al., 2020), the effect of melanogenesis on photosynthesis was expected, and one can assume that plastid reorganization at the late milk stage (when chloroplasts turn into melanoplasts) causes the observed suppression of expression of the genes related to photosynthetic activity in the melanin-accumulating grain. At the same time, in the BP line, which accumulates anthocyanins and melanins, such underexpression of photosynthesis genes was not detectable at any stages. What may be related to the protective effect of anthocyanins on photosynthetic apparatus is their antioxidant capacity, which can defend tissues against the reactive oxygen species generated in the chloroplast (Guidi et al., 2016; Mushtaq et al., 2016). In the melanin-accumulating lines downregulation of the photosynthesis-related genes was accompanied by intensification of the fatty acid biosynthesis and lipid biosynthetic process at later developmental stages. These processes may occur in melanoplasts where synthesis of melanin takes place. However, in opposite to another type of aging plastids — gerontoplasts —, where degradation of thylakoid lipids and releasing the fatty acids have been observed (Biswal et al., 2003), in melanoplasts, active biogenesis of these chemicals that are components of cell membranes, including thylakoids, may take place for melanin sequestration in cells. Indeed, the presence of membrane-delimited structures in melanin-accumulating lines was observed up to the hard-dough stage when the plastids were degraded totally in non-pigmented lines (Mursalimov et al., 2022).

## Conclusion

Here, with the help of NILs as a genetic model, a significant impact of melanogenesis on metabolic processes that take place in barley grain was revealed. Downregulation of the photosynthesis-related genes, accompanied by intensification of the fatty acid biosynthesis, lipid biosynthetic processes and significant changes in the phenolic metabolism was showed in the melanin-accumulating lines. These lines showed an increased total level of benzoic acids, among them, protocatechuic and vanillic acids were identified. By contrast, anthocyanin-accumulating lines showed higher concentrations of flavonoids and *p*-coumaric and ferulic acids. Based on the data the simple phenolic compounds that synthesized in plastids where melanin synthesis occurs too were suggested as likely precursors of melanin in barley.

## Data Availability Statement

The datasets presented in this study can be found in online repositories. The names of the repository/repositories and accession number(s) can be found below: https://www.ncbi.nlm.nih.gov/, PRJNA832149.

## Author Contributions

AG contributed to interpretation of the data and study conception and wrote the original draft of the manuscript. AV and NAS performed the analysis of the transcriptomic data and participated in the drafting of the manuscript. SM and EC performed the extraction and analysis of phenolic compounds and participated in the drafting of the manuscript. GV and NVS prepared the RNA-seq libraries and carried out the sequencing of the libraries on the Illumina platform. EK participated in interpretation of the data and revised the manuscript critically. OS conceived the study and contributed to its design and coordination, interpretation of the data, and manuscript writing. All authors reviewed and edited the manuscript.

## Conflict of Interest

The authors declare that the research was conducted in the absence of any commercial or financial relationships that could be construed as a potential conflict of interest.

## Publisher’s Note

All claims expressed in this article are solely those of the authors and do not necessarily represent those of their affiliated organizations, or those of the publisher, the editors and the reviewers. Any product that may be evaluated in this article, or claim that may be made by its manufacturer, is not guaranteed or endorsed by the publisher.

## Footnotes

1
www.nordgen.org

2
http://plants.ensembl.org

3
http://www.lifetechnologies.com
