# Supplementary material for: Features of Activity of the Phenylpropanoid Biosynthesis Pathway in Melanin-Accumulating Barley Grains
Source: Front Plant Sci. 2022 Jul 11;13:923717. doi: 10.3389/fpls.2022.923717 (PMC9310326; doi:10.3389/fpls.2022.923717)
Supplement: Supplementary file 9 [file Data_Sheet_3.PDF]

Supplementary Material

Supplementary Figure 1. Venn diagrams of upregulated and downregulated DEGs between lines BLP, PLP, BP and BW (Bowman) at the stage 1.

Supplementary Figure 2. Venn diagrams of upregulated and downregulated DEGs between lines BLP, PLP, BP and BW (Bowman) at the stage 2.

Supplementary Figure 3. Venn diagrams of upregulated and downregulated DEGs between lines BLP, PLP, BP and BW (Bowman) at the stage 3.

Supplementary Table 7. Number of DEGs between the NILs within stages 1, 2 and 3. FDR<0,05.

Supplementary Figure 4. HPLC chromatographic profiles of hydroxycinnamic acids at 25 °C. The assignment of numbered peaks is shown in Supplementary Table 6.

Supplementary Figure 5. HPLC chromatographic profiles of flavonoids at 25 °C. The assignment of numbered peaks is shown in Supplementary Table 6.

Supplementary Figure 6. HPLC chromatographic profiles of anthocyanins at 25 °C. The assignment of numbered peaks is shown in Supplementary Table 6.

Supplementary Figure 7. HPLC chromatographic profile of one of the mixtures of standard compounds at 25 °C: 1 - 3,4-dihydroxybenzoic acid, 2 – catechin, 3 – dihydromyricetin, 4 - vanillic acid, 5 - trans-caffeic acid, 6 - 4-hydroxycinnamic acid, 7 – dihydroquercetin, 8 - trans-ferulic acid, 9 - dimethoxybenzoic acid, 10 - 3-hydroxycinnamic acid, 11 – dihydrokaempferol, 12 – hesperidin, 13 - rutin, trihydrate, 14 - 2-hydroxycinnamic acid, 15 - cinnamic acid, 16 – quercetin, 17 – naringenin.

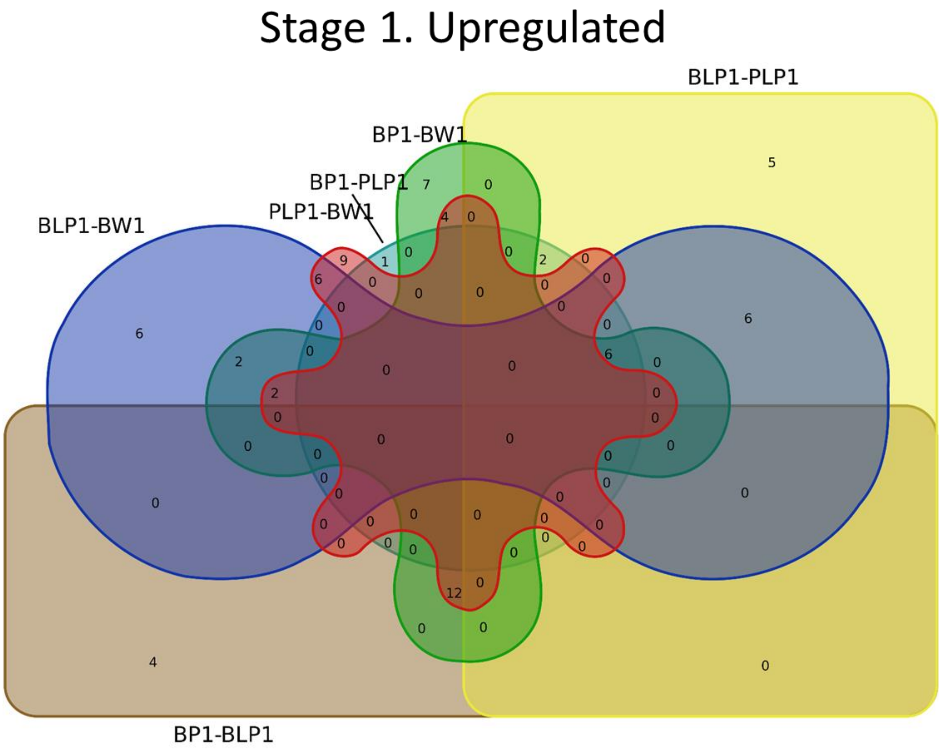

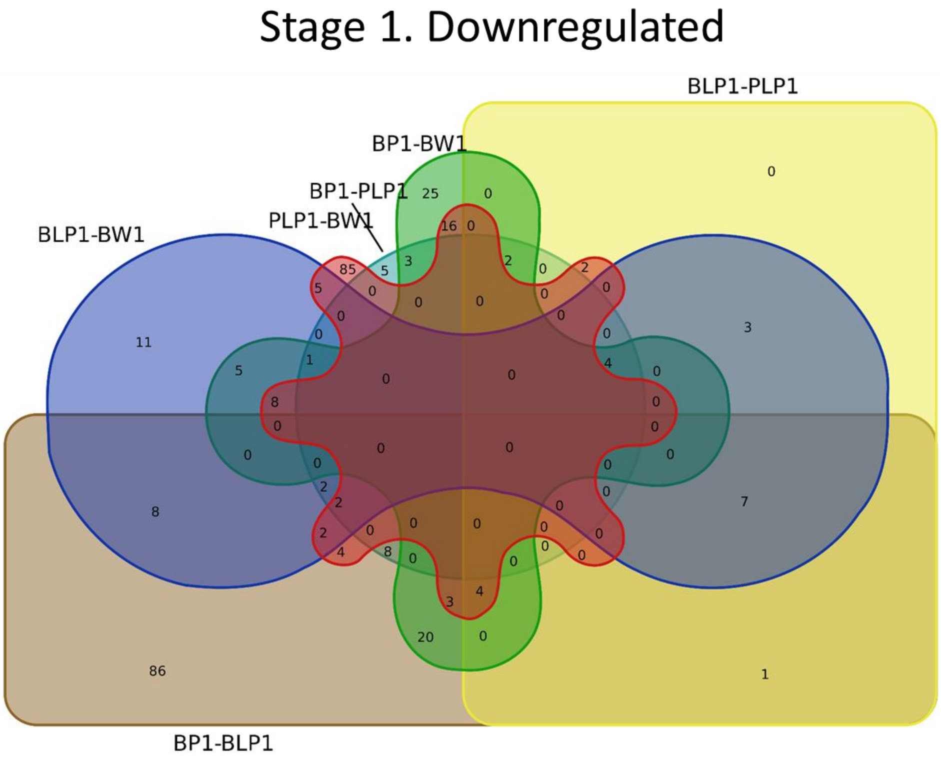

Supplementary Figure 1. Venn diagrams of upregulated and downregulated DEGs between lines BLP, PLP, BP and BW (Bowman) at the stage 1.

Stage 2. Upregulated

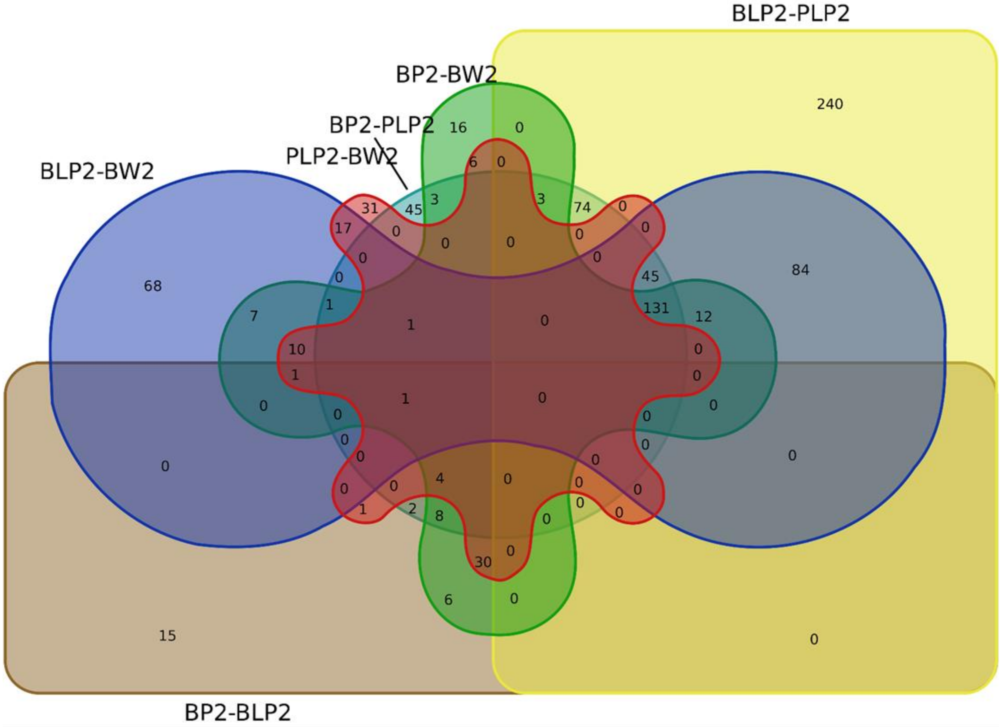

Stage 2. Downregulated

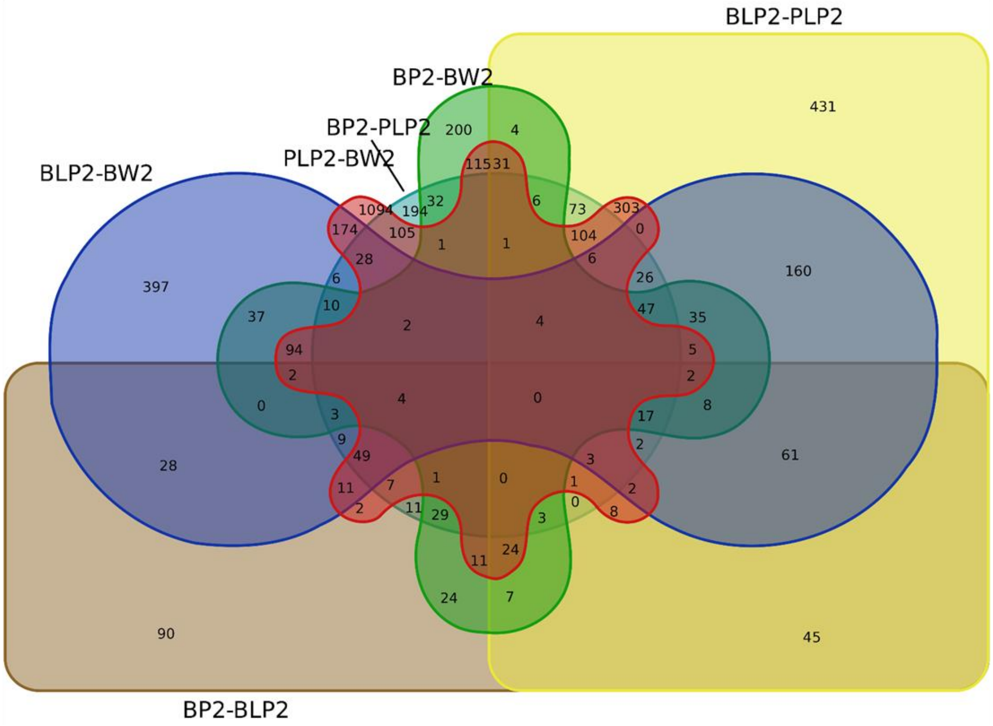

Supplementary Figure 2. Venn diagrams of upregulated and downregulated DEGs between lines BLP, PLP, BP and BW (Bowman) at the stage 2.

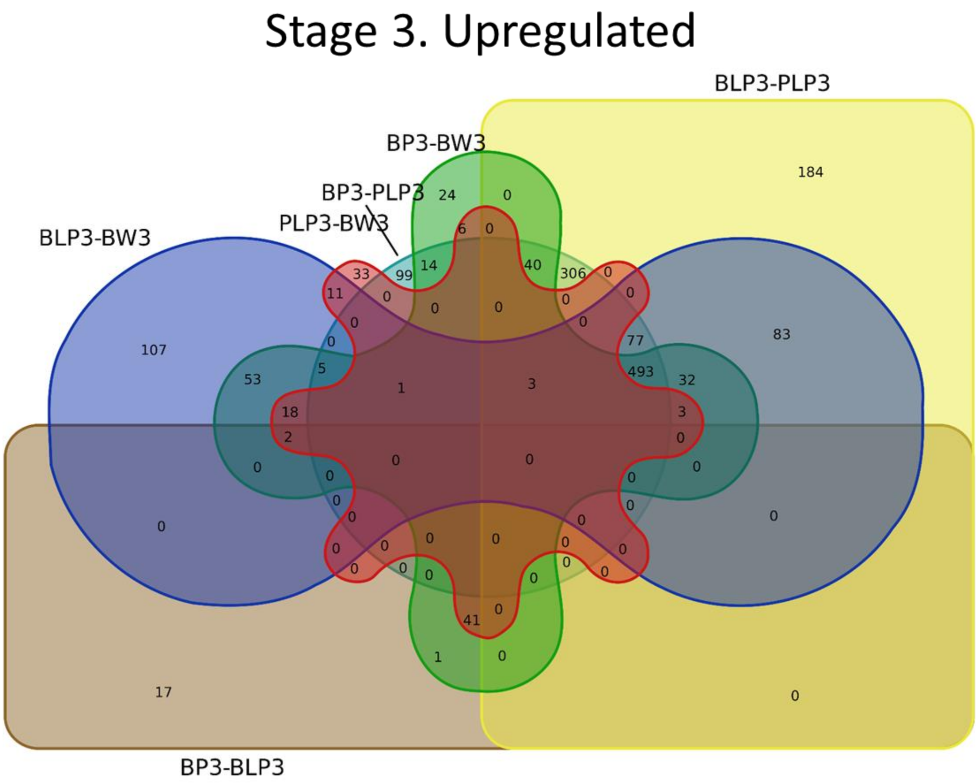

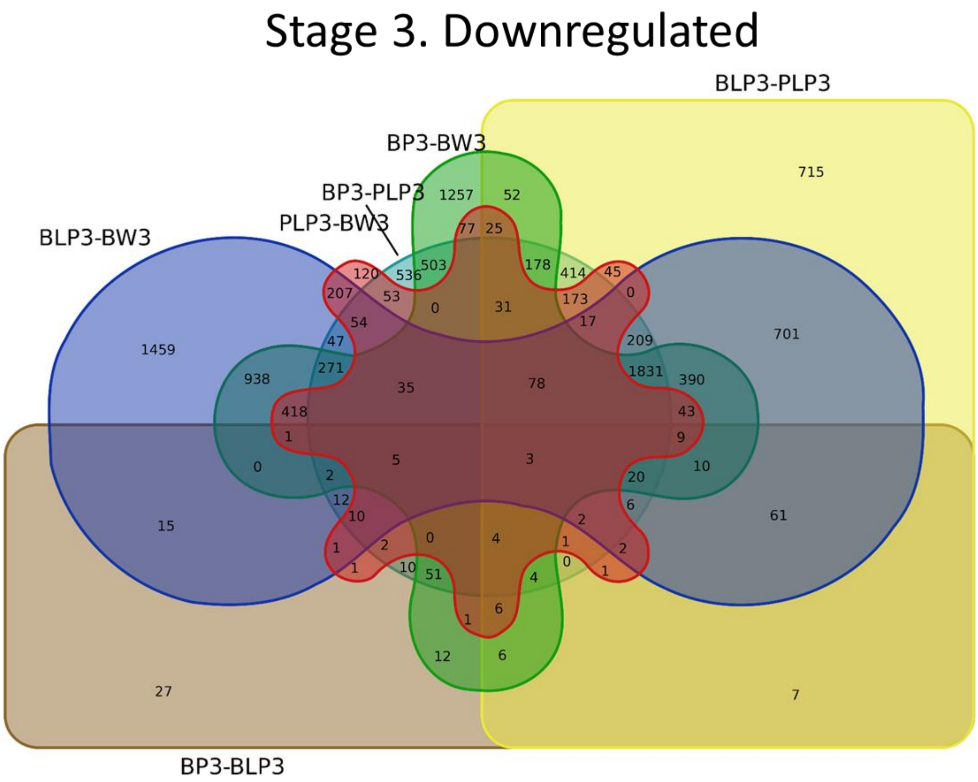

Supplementary Figure 3. Venn diagrams of upregulated and downregulated DEGs between lines BLP, PLP, BP and BW (Bowman) at the stage 3.

Supplementary Table 7. Number of DEGs between the NILs within stages 1, 2 and 3. FDR<0,05.

Number of upregulated DEGs						
	BLP vs. BW	PLP vs. BW	BP vs. BW	BLP vs. PLP	BP vs. BLP	BP vs. PLP
Stage 1	28	33	33	19	16	9
Stage 2	378	102	240	589	68	318
Stage 3	888	118	736	1221	61	1038
Number of downregulated DEGs						
	BLP vs. BW	PLP vs. BW	BP vs. BW	BLP vs. PLP	BP vs. BLP	BP vs. PLP
Stage 1	21	195	52	19	22	17
Stage 2	222	358	70	59	49	20
Stage 3	426	226	570	283	117	200

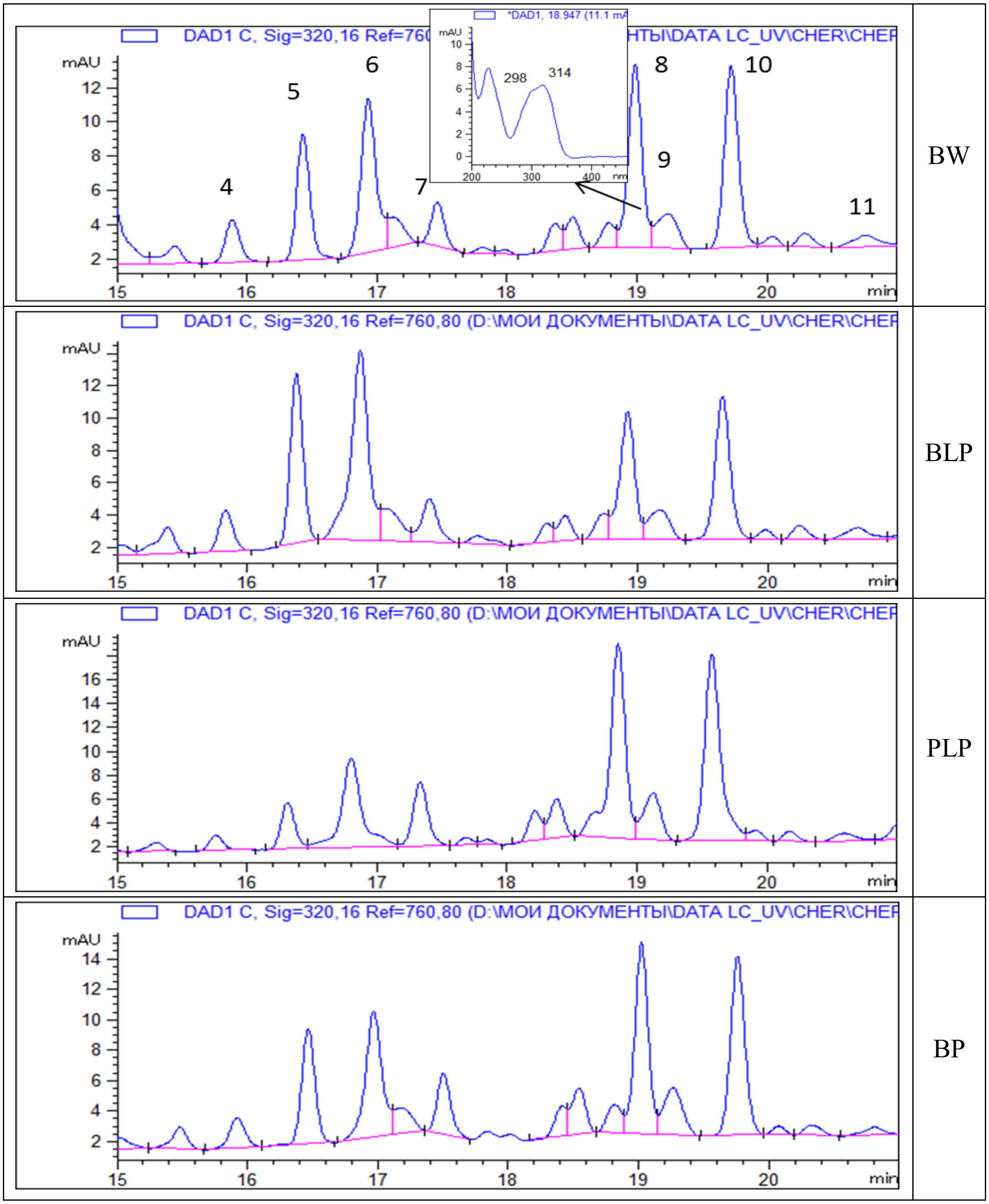

Supplementary Figure 4. HPLC chromatographic profiles of hydroxycinnamic acids at 25 °C. The assignment of numbered peaks is shown in Supplementary Table 6.

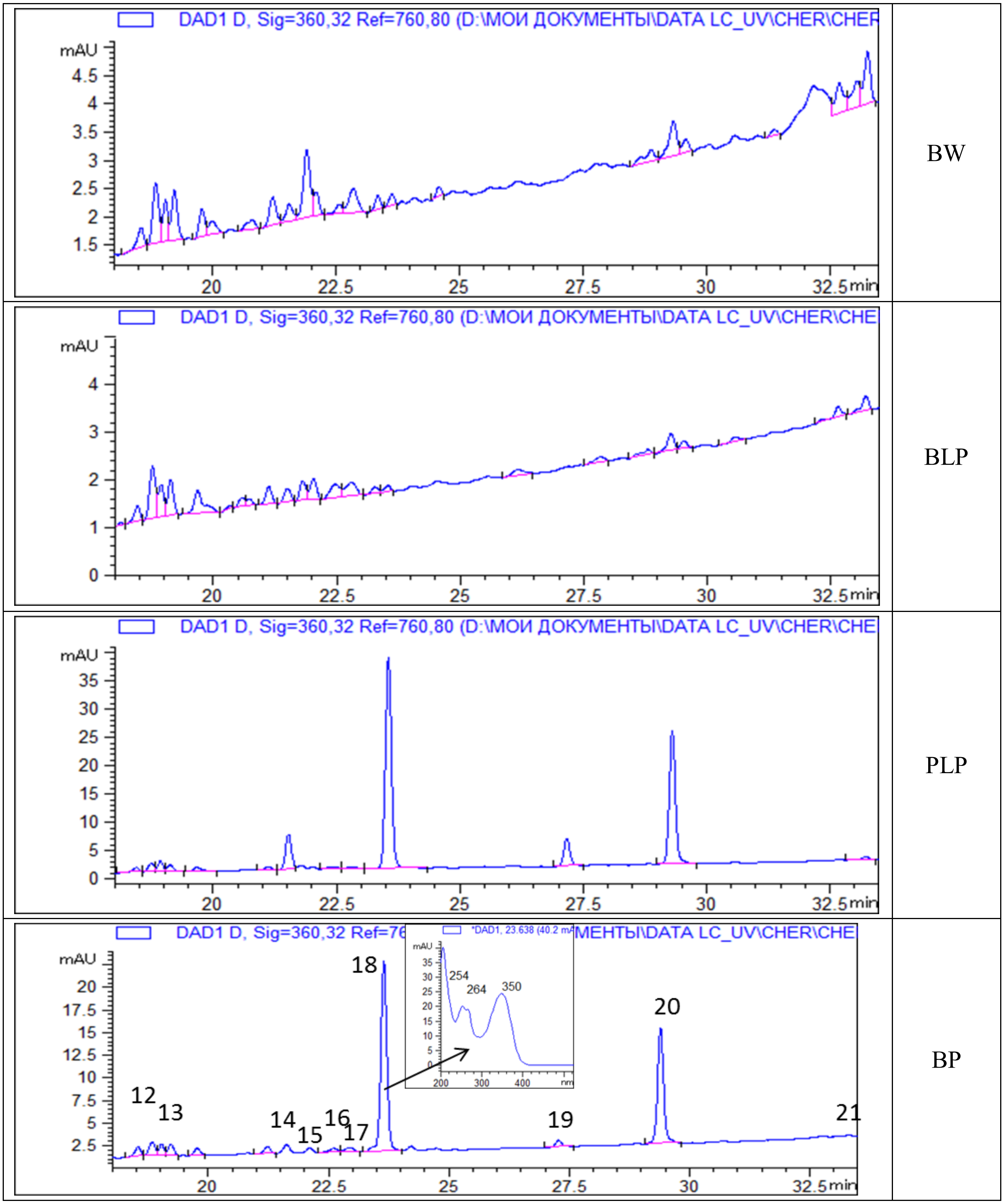

Supplementary Figure 5. HPLC chromatographic profiles of flavonoids at 25 °C. The assignment of numbered peaks is shown in Supplementary Table 6.

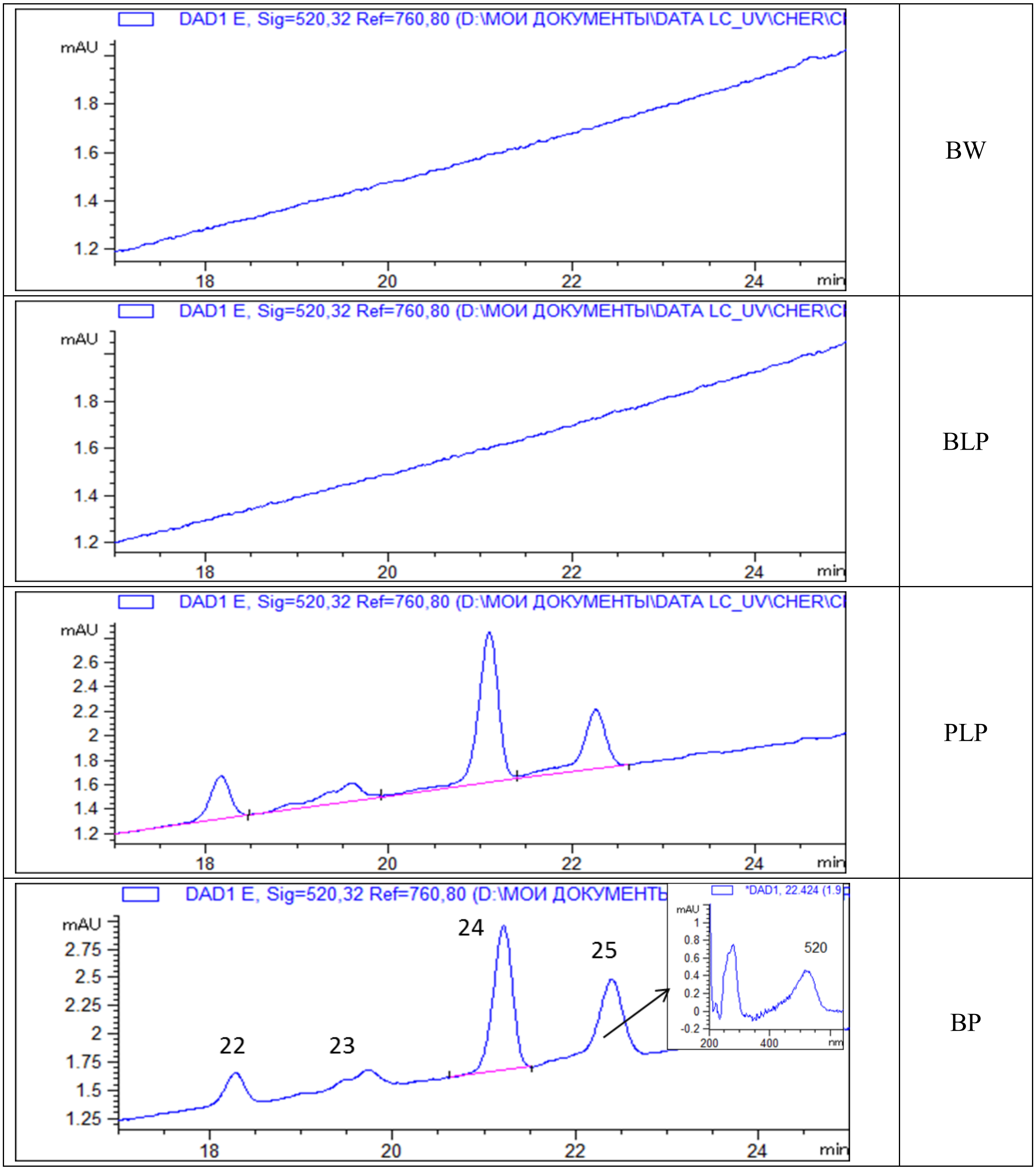

Supplementary Figure 6. HPLC chromatographic profiles of anthocyanins at 25 °C. The assignment of numbered peaks is shown in Supplementary Table 6.

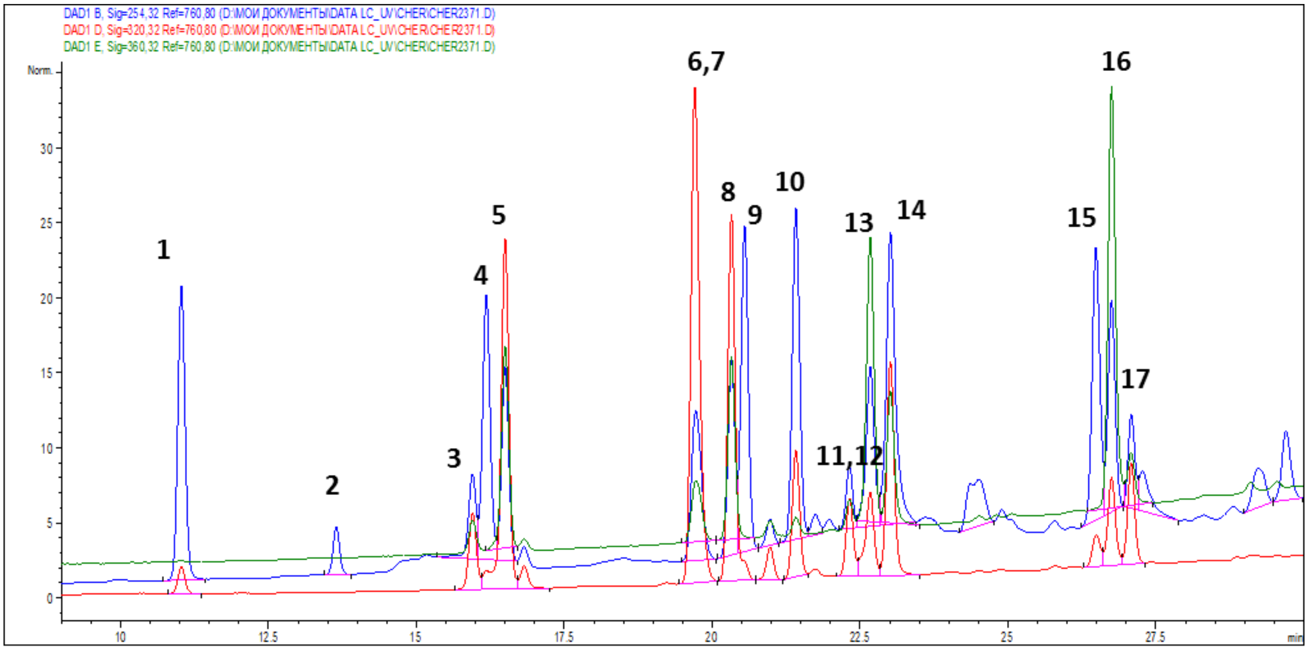

Supplementary Figure 7. HPLC chromatographic profile of one of the mixtures of standard compounds at 25 °C: 1 - 3,4-dihydroxybenzoic acid, 2 – catechin, 3 – dihydromyricetin, 4 - vanillic acid, 5 - trans-caffeic acid, 6 - 4-hydroxycinnamic acid, 7 – dihydroquercetin, 8 - trans-ferulic acid, 9 - dimethoxybenzoic acid, 10 - 3-hydroxycinnamic acid, 11 – dihydrokaempferol, 12 – hesperidin, 13 - rutin, trihydrate, 14 - 2-hydroxycinnamic acid, 15 - cinnamic acid, 16 – quercetin, 17 – naringenin.
